# SWAT Modeling of Non-Point Source Pollution in Depression-Dominated Basins under Varying Hydroclimatic Conditions

**DOI:** 10.3390/ijerph15112492

**Published:** 2018-11-08

**Authors:** Mohsen Tahmasebi Nasab, Kendall Grimm, Mohammad Hadi Bazrkar, Lan Zeng, Afshin Shabani, Xiaodong Zhang, Xuefeng Chu

**Affiliations:** 1 Department of Civil and Environmental Engineering (Dept 2470), North Dakota State University, PO Box 6050, Fargo, ND 58108-6050, USA; m.tahmasebinasab@ndsu.edu (M.T.N.); kendall.grimm@ndsu.edu (K.G.), mohammadhadi.bazrkar@ndsu.edu (M.H.B.); lan.zeng@ndsu.edu (L.Z.); 2 Department of Earth System Science & Policy, University of North Dakota, 4149 University Ave Stop 9011, Grand Forks, ND 58202-6089, USA; afshin.shabani@und.edu (A.S.); xiaodong.zhang2@und.edu (X.Z.)

**Keywords:** SWAT, hydrologic modeling, water quality modeling, depressions, watershed

## Abstract

Non-point source (NPS) pollution from agricultural lands is the leading cause of various water quality problems across the United States. Particularly, surface depressions often alter the releasing patterns of NPS pollutants into the environment. However, most commonly-used hydrologic models may not be applicable to such depression-dominated regions. The objective of this study is to improve water quantity/quality modeling and its calibration for depression-dominated basins under wet and dry hydroclimatic conditions. Specifically, the Soil and Water Assessment Tool (SWAT) was applied for hydrologic and water quality modeling in the Red River of the North Basin (RRB). Surface depressions across the RRB were incorporated into the model by employing a surface delineation method and the impacts of depressions were evaluated for two modeling scenarios, MS1 (basic scenario) and MS2 (depression-oriented scenario). Moreover, a traditional calibration scheme (CS1) was compared to a wet-dry calibration scheme (CS2) that accounted for the effects of hydroclimatic variations on hydrologic and water quality modeling. Results indicated that the surface runoff simulation and the associated water quality modeling were improved when topographic characteristics of depressions were incorporated into the model (MS2). The Nash–Sutcliffe efficiency (NSE) coefficient indicated an average increase of 30.4% and 19.6% from CS1 to CS2 for the calibration and validation periods, respectively. Additionally, the CS2 provided acceptable simulations of water quality, with the NSE values of 0.50 and 0.74 for calibration and validation periods, respectively. These results highlight the enhanced capability of the proposed approach for simulating water quantity and quality for depression-dominated basins under the influence of varying hydroclimatic conditions.

## 1. Introduction

Agricultural activities are the major source of non-point source (NPS) pollution in the United States [1,2,3]. When the NPS pollutants enter the aquatic ecosystem, they impair water resources and bring about many environmental problems and challenges such as eutrophication, loss of biodiversity, and oxygen depletion. In the United States, for example, 60% of the impaired stream reaches and almost 50% of the impaired lakes are ascribed to the destructive effects of eutrophication [2]. Since NPS pollution is closely associated with time-dependent agricultural activities (e.g., applications of fertilizers and pesticides) and hydrologic processes (e.g., precipitation and surface runoff), NPS pollution is difficult to measure and control [1]. A large body of literature addressing NPS pollution in the United States was developed in the 1970s and 1980s [4]. Specifically, the U.S. Congress amended the Clean Water Act (CWA) in 1987 to establish the Section 319 NPS Management Program, under which the success of specific NPS implementation projects was assessed [5]. Although the national NPS program has succeeded in some aspects, agricultural NPS pollution is still a growing concern in the United States [6] and more actions are needed to both restore the NPS-impaired water bodies as well as protect the unimpaired water bodies from NPS pollution [7].

Hydrologic and water quality models have been historically developed and utilized to simulate hydrologic processes as well as the fate and transport of various NPS contaminants in basins [8,9]. The Soil and Water Assessment Tool (SWAT) [10,11], Hydrological Simulation Program-Fortran (HSPF) [12], Annualized Agricultural Non-Point Source model (AnnAGNPS) [13], and many other models have been developed and widely used for watershed-scale water quantity and quality modeling under a variety of climatic and hydrologic conditions around the world, e.g., [14,15,16]. However, such models may not be applicable to depression-dominated regions due to their intrinsic limitations. Surface depressions are essential topographic features that play an influential role in overland flow generation and runoff processes and, as a result, basin response [17,18,19]. These topographic features act like “gatekeepers” within a basin [17]. In other words, they keep storing water from their contributing areas until their ponded water reaches their maximum storage levels. Only when these depressions are fully filled and connected to the drainage network do they contribute runoff to the basin outlet. This physical process is particularly accentuated for depression-dominated regions. Despite the significance of depressions on hydrologic processes, delineation of these depressions and modeling of flow and contaminant transport associated with these depressions have been a challenge.

While various hydrography inventories, such as the United States Geological Survey (USGS) National Hydrography Dataset (NHD), and remote sensing technologies have improved the mapping and monitoring of surface depressions, quantifying the distribution of depressions across a basin is still a problematic task due to their temporal changes. Although available datasets provide the spatial distribution of depressions across a surface, they do not provide any additional information on the storage and variations of the identified depressions, which are critical to performing physically-based hydrologic modeling, specifically for depression-dominated basins. As a result, some attempts have been made to quantify depressions by delineating surface depressions using digital elevation models (DEMs) (e.g., [20,21,22]). For example, Tahmasebi Nasab et al. [22] developed a new Depression-Dominated Delineation (D-cubed) method based on the Puddle Delineation (PD) algorithm [21] to identify depressions and quantify their detailed topographic characteristics such as maximum depression storage (MDS) and contributing area. Many studies have incorporated the results of delineation methods to investigate the impacts of depressions on the surface and subsurface processes (e.g., [17,18,23,24,25,26,27]). Chu et al. [25] developed the Puddle-to-Puddle (P2P) hydrologic modeling system to simulate the detailed depression filling, spilling, merging, and splitting dynamics across space and time. Tahmasebi Nasab et al. [17] coupled the PD algorithm [21] with the SWAT model [10,11] to incorporate topographic characteristics of depressions into hydrologic modeling of the Upper-Pipestem basin, North Dakota. In another SWAT study, Mekonnen et al. [27] utilized a probability-distributed model of depression storage to account for depression storage heterogeneity across two basins in Canada.

Annual precipitation and other climatic variables determine the overall hydroclimatic conditions (wet or dry years) and directly affect streamflow in a basin. Such hydroclimatic variations are intensified in basins with unique topographic and hydrologic characteristics. For instance, the flat topography, fine-textured soils, and long cold winters in the Red River of the North Basin (RRB) are the major factors that induce the spring floods [28]. The annual variations in streamflow have been studied from different aspects (e.g., [29,30]). Li et al. [29] investigated the impacts of climate variability and human activities on annual streamflow changes for a 37-year period in the Wuding River basin, China. It was concluded that the soil conservation measures in Wuding River basin were one of the key factors in reducing the streamflow variability [29]. In another study, Gao et al. [30] assessed the impacts of separated calibrations for wet and dry years in the Barrett watershed, southern California, on the SWAT model efficiency, and found that separating the simulation period into wet and dry years improved the modeling efficiency.

The objective of this study is to improve hydrologic and NPS water quality modeling and calibration for depression-dominated basins under wet and dry hydroclimatic conditions. To accomplish this objective, the SWAT model is employed to perform water quantity and quality modeling for the RRB, which is characterized by the presence of numerous depressions. Depressions over the RRB are identified by using a DEM-based delineation method. Two SWAT modeling scenarios, MS1 (basic scenario without incorporating surface depressions) and MS2 (depression-oriented scenario), are developed to evaluate the impacts of surface depressions on the hydrologic processes and the fate of nutrients. Lastly, the effects of hydroclimatic variations on hydrologic and water quality modeling are investigated with two calibration schemes, CS1 (traditional scheme) and CS2 (separated wet and dry years scheme).

## 2. Materials and Methods

### 2.1. Study Area

**Figure 1:**
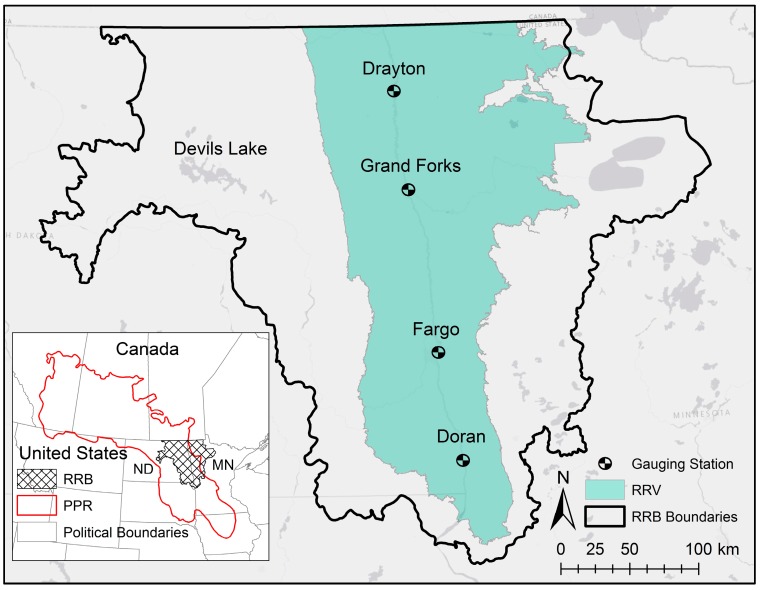
Prairie Pothole Region (PPR), Red River of the North Basin (RRB), Red River Valley (RRV), and four gauging stations in Doran, Fargo, Grand Forks, and Drayton.

The prairie pothole region (PPR), covering parts of five U.S. states and three Canadian provinces, is characterized by numerous pothole wetlands and depressions, which affect hydrologic processes, nutrient retention, and occurrence of natural floods [31]. Although the potholes in the PPR are mostly isolated, they can potentially spill over their thresholds when they are fully filled and contribute surface runoff to their downstream areas [19,32,33]. In this study, we selected the U.S. part of the RRB that drains 103,600 km^2^ of three U.S. states [34] (Figure 1). Following Lin et. al. [28], the Devils Lake watershed, which is a terminal basin, was excluded from the study area.

The slopes of the RRB range from 0.04 to 0.25 m/km [28]. Specifically, the central part of RRB (i.e., the Red River Valley (RRV)), which used to be the bottom of glacial Lake Agassiz, possesses a flat topography. The soil type, also affected by the bottom of former glacial Lake Agassiz, is mainly comprised of lacustrine soil [35,36]. The average precipitation and average annual temperature in the RRB are 500 mm and 4.3 °C, respectively [28,35]. The basin receives a majority of its rainfall from mid-May to mid-September and, therefore, the RRB is subject to flooding due to snowmelt in early spring. Three historical floods in RRB took place in 1997, 2009, and 2011, out of which the 2009 flood ranks among the highest floods ever recorded in North Dakota [37]. These frequent spring floods lead to annual streamflow variabilities and make it difficult for hydrologic models to provide reliable estimates of streamflow for both wet and dry years.

The fertile RRV also plays an important role in North Dakota agriculture. According to the National Agricultural Statistics Service, the Cropland Data Layer (CDL) Program [38], only 4.5% of the RRB was covered by developed areas in 2017. Soybean, spring wheat, and corn were the dominant crops and had the highest acreage in 2017 across the RRB. Application of fertilizers and pesticides to the agricultural fields in the RRB can adversely affect its aquatic systems. Previous studies in the RRB have highlighted the important influences of agricultural activities on the quality of the basin’s water (e.g., [28,36,39]). For example, Stoner et al. [39] found that the tributaries of the Red River, which are within the regions of extensive agricultural activities (mainly in the RRV), had the highest concentrations of nutrients (dissolved phosphorus, nitrate, and organic nitrogen). Lin et al. [28] investigated the agricultural land use change in the RRB and evaluated the impacts of these changes on water quantity and quality of the Red River. However, topographic depressions and their associated impacts have not been investigated in those studies.

### 2.2. Water Quantity and Quality Modeling

The SWAT model [10,11] was used in this study to perform water quantity and quality modeling for the RRB. SWAT is a process-based hydrologic model designed to evaluate the impacts of land use and management on water, sediments, and pollutants in agricultural basins [40]. In SWAT, the basic modeling unit is the Hydrologic Response Unit (HRU), which is a homogeneous combination of land use, topography, and soil features. SWAT simulations are provided in the land phase and routing phase [40]. In other words, water and pollutants, flowing to the main channel, are first simulated for each subbasin. Then, they are routed to the outlet of the basin. The plant growth model provides estimates of water and nutrient uptake from the root zone and predicts transpiration and yield production of specific crops. In SWAT, impounded water can be simulated in different forms: (1) ponds, (2) wetlands, (3) depressions/potholes, and (4) reservoirs; each of which has unique features [40]. For instance, ponds and wetlands are incorporated within subbasins, whereas depressions/potholes and reservoirs are simulated within HRUs and in the main channel network, respectively. These forms of impounded water in SWAT have been used in different studies (e.g., [17,26,41]). For example, Wang et al. [26] incorporated wetlands into SWAT using a Hydrologic Equivalent Wetland (HEW) concept and compared their results with other scenarios assuming no wetland and synthetic wetland.

#### 2.2.1. Model Development

The SWAT model for the RRB was developed using different datasets. Daily meteorological data (i.e., precipitation and temperature) were obtained from the Climate Forecast System Reanalysis (CFSR) dataset [42]. A 30-m resolution DEM of the study area was downloaded from the U.S. Geological Survey [43] and used for surface delineation. A simple DEM-based delineation method developed by Planchon and Darboux [20] was utilized in this study to estimate the potential depression storage and the ArcPy package [44] in Python 2.7 scripting language [45] was used to automate this delineation process. To account for the detailed land use across the RRB, the United States Department of Agriculture (USDA)’s Cropland Data Layer (CDL) [38] was downloaded for land use and land cover information. Also, the State Soil Geographic (STATSGO) dataset [46] was used to provide the model with the soil classifications. Thirty-year (1988–2017) daily streamflow data were obtained for four USGS gauging stations along the Red River (Doran, Fargo, Grand Forks, and Drayton stations) (Figure 1) and the discharge data were analyzed. Nitrate-nitrogen (NO_3_-N) concentrations irregularly measured at the Grand Forks gauging station were also obtained from USGS. To convert these concentration data into monthly loading, a regression analysis was performed for different months, relating the observed discharge to NO_3_-N loading. Four RRB’s major crops (i.e., corn, soybean, spring wheat, and sugar beet) were selected for crop modeling and fertilizer application using the North Dakota fertilizer recommendation tables [47]. A simulation period of 24 years (1989–2012) was selected, out of which the initial 5 years (1989–1993) were used as the model warm-up period; the 1994–2003 period was specified for model calibration; and the 2004–2012 period was specified for model validation. This selected 24-year simulation period is characterized by the occurrence of both wet and dry years to highlight the roles of hydroclimatic variations in the model calibration process. To separate the calibration and validation periods, the relative deviation percentage from the mean streamflow was used to ensure that both wet and dry years existed in both calibration and validation periods.

#### 2.2.2. Modeling Scenarios and Calibration Schemes

Two modeling scenarios, MS1 (basic scenario) and MS2 (depression-oriented scenario), were considered in this study to evaluate the effects of surface depressions on SWAT hydrologic and water quality modeling. MS1 involved basic SWAT modeling without incorporating depressions, whereas MS2 incorporated the delineated depressions into the simulation. The maximum depression storage (WET_MXVOL) and maximum ponding area (WET_MXSA) delineated for each subbasin were applied to the SWAT wetland feature, which received water from a fraction of each subbasin (WET_FR) [40]. The WET_FR of a subbasin was the ratio of the maximum ponding area to the subbasin area. Simulation results from MS1 and MS2 were compared for the depression-dominated and non-depressional subbasins to evaluate the impacts of depressions on the simulated hydrologic processes. MS2 was chosen to evaluate different calibration schemes.

**Figure 2:**
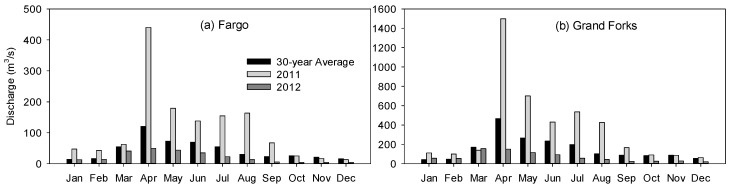
Monthly average discharges for 2011, 2012, and a 30-year period (1988–2017) at (**a**) Fargo station, and (**b**) Grand Forks station.

Studies have shown that SWAT failed to simulate flood peaks in the RRB [48]. This issue can be attributed to the snowmelt and hydroclimatic variations in the RRB in wet and dry years. Streamflow analysis for a 30-year period (1988–2017) at the Fargo station shows a distinct trend of annual and inter-annual streamflow variations (Figure 2a). This trend can also be observed at other stations in the RRB. For example, for the same 30-year period at the Grand Forks station, 63.34% of the monthly average peak flows occurred during April, May, and June (Figure 2b). A combination of the inter-annual variability and climate variations led to annual streamflow variations in the Red River. Figure 2 illustrates the inter-annual variations of streamflow at the Fargo station (Figure 2a) and the Grand Forks station (Figure 2b), showing significant annual streamflow variations in 2011 and 2012. From Figure 2a, it can be observed that the months from March to August accounted for all major high flows. This can be attributed to the unique hydrologic characteristics of the RRB, where the snowmelt process during March and April overlaps the rainfall period. Figure 2b also shows a similar trend at the Grand Forks station. However, different years, depending on their hydroclimatic conditions, can be wet or dry. Note that the streamflow in 2011 is noticeably higher than the 30-year average, while the streamflow in 2012 is much lower than the 30-year average (Figure 2). The annual streamflow variations can also be observed for other years and other stations in the RRB.

In this study, two calibration schemes, CS1 (traditional calibration scheme) and CS2 (wet-dry calibration scheme), were conducted. CS1 followed the traditional approach for model calibration and validation over the entire simulation period. CS2 took into account the annual variations of streamflow and implemented separate model calibrations for wet and dry years that were determined based on the relative deviation percentage from the mean streamflow [30]. Thus, in CS2, the years with similar hydroclimatic conditions (wet or dry) were grouped together to ensure a uniform calibration process for these two distinct conditions. Table 1 presents the wet and dry years for the four gauging stations considered in this study.

The SUFI2 optimization algorithm implemented in SWAT Calibration and Uncertainty Procedures (SWAT-CUP) 2012 [49] was used for conducting the calibration process. The water quantity modeling was calibrated by using the observed discharges at the four USGS gauging stations in Doran, MN; Fargo, ND; Grand Forks, ND; and Drayton, ND (Figure 1), while the water quality modeling was calibrated by using the observed NO_3_-N loads at the Grand Forks station. In CS2, the wet and dry years were calibrated separately. To compare the two calibration schemes, the streamflow and nutrient calibration parameters and their initial ranges were kept the same for both CS1 and CS2. The model was then validated for both traditional and wet-dry schemes. The goodness of fit for each scheme was evaluated by using two recommended statistics for streamflow and nutrient loads [50,51]: (1) Nash-Sutcliffe efficiency (NSE) and (2) percent bias (PBIAS). The criteria recommended for satisfactory model performance were: NSE > 0.50 [50,51] and PBIAS = ±15% for streamflow [51] and PBIAS = ±30% for nutrients [51]. NSE [52] and PBIAS are respectively given by:

(1) $$NSE=1-\frac{\sum_{i=1}^{n}{\left(Y_{i}^{obs}-Y_{i}^{sim}\right)}^{2}}{\sum_{i=1}^{n}{\left(Y_{i}^{obs}-{\bar{Y}}^{obs}\right)}^{2}}$$

(2) $$PBIAS=\frac{\sum_{i=1}^{n}(Y_{i}^{obs}-Y_{i}^{sim})\times 100}{\sum_{i=1}^{n}Y_{i}^{obs}}$$

where $Y_{i}^{obs}$ is the *i*th observed value, $Y_{i}^{sim}$ is the *i*th simulated value, ${\bar{Y}}^{obs}$ is the mean of the observed values, and *n* is the total number of observations.

## 3. Results

### 3.1. Watershed Delineation and Depression Storage

**Figure 3:**
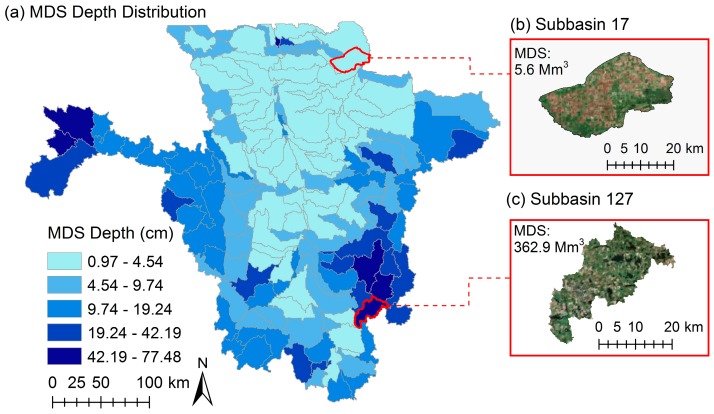
(**a**) Distribution of maximum depression storage (MDS) depths over the study area and two selected subbasins with (**b**) minimum MDS, and (**c**) maximum MDS.

The study area was divided into 146 subbasins ranging from 5.08 km^2^ to 2320 km^2^. Based on the land use, soil type, and slope thresholds of 10%, 10%, and 20%, respectively, 1407 HRUs were defined for the model. The surface topographic delineation results indicated that the MDS over the entire study area was 10.7 cm. Figure 3a depicts the distribution of MDS depths (MDS/subbasin area) for different subbasins in the study area. From Figure 3a, it can be observed that the central part of the RRB (i.e., RRV) generally accounts for lower MDS values. MDS increases from the central part to the eastern and western sides of the study area (Figure 3a). Subbasins 17 and 127 have the minimum and maximum MDS values (Figure 3b,c), respectively. A visual comparison of the land covers between these two subbasins reveals the depression-dominated nature of subbasin 127, whereas subbasin 17 is dominated by agricultural fields. In addition, the land cover distribution shows that subbasin 17 contains no open water, whereas 17% of subbasin 127 (77.8 km^2^) is covered by water (Figure 3b,c). Subbasins 17 and 127 have the MDS depths of 0.97 cm and 77.48 cm, and the maximum ponding areas of 58 km^2^ and 219 km^2^, respectively. These results are in accordance with other studies in the RRB. For example, Ludden et al. [53] estimated the maximum depression storage of depressions in the Devils Lake watershed in North Dakota by using the photogrammetric technique and found that the MDS depth of the Devils Lake watershed was 53 cm.

### 3.2. How Do Depressions Alter Modeling Results?

To determine the impacts of depressions on the modeling of hydrologic processes, the simulated surface runoff for the two modeling scenarios (MS1 and MS2) were compared for the two selected subbasins, subbasins 127 and 17, which respectively represented depression-dominated and non-depressional subbasins. The average discrepancy ratio of the simulated surface runoff in subbasin 17 for MS1 and MS2 was 2.4. In other words, the simulated surface runoff in MS1 was on average 2.4 times greater than that in MS2. This discrepancy can be directly attributed to the amount of water stored in the depressions of subbasin 17. The impact of depressions on surface runoff is even more notable in subbasin 127, where its sizable depression storage led to a large discrepancy ratio (7.5) in the simulated surface runoff values between MS1 and MS2. This significant difference again demonstrates the role of depressions as “gatekeepers” and their effects on the generation of surface runoff in different subbasins. This finding is consistent with other studies (e.g., [17,27]).

**Figure 4:**
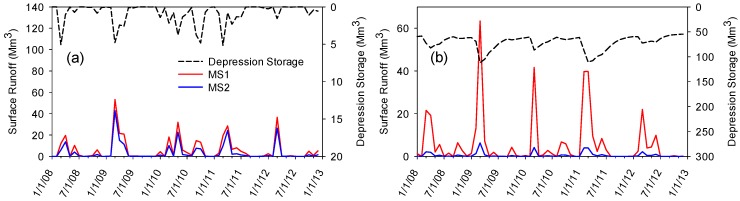
Simulated monthly surface runoff and depression storage of (**a**) subbasin 17 and (**b**) subbasin 127 in a selected time period (2008–2013) for modeling scenarios MS1 and MS2.

To better analyze the discrepancies in the two selected subbasins, Figure 4 compares the simulated surface runoff and depression storage for a selected simulation period from January 2008 to December 2012. From Figure 4a,b, it is noteworthy to observe that MS1 resulted in consistently higher surface runoff values in both subbasins. However, the variations in surface runoff from MS1 to MS2 were less pronounced in subbasin 17 (Figure 4a). Figure 4a also shows sudden fluctuations of depression storage in subbasin 17 over the course of the selected simulation period. The results indicated that when the surface runoff increased, a sharp rise was observed in the corresponding depression storage in subbasin 17. These sharp rises were immediately followed by sudden drops in the amount of depression storage. This stepwise rising-falling trend, which can be attributed to the lack of major depressions of subbasin 17, was also detected for the rest of the simulation period. However, the depression storage in subbasin 127 never experienced a dry condition (Figure 4b). The average depression storage of subbasin 127 during the entire simulation period is 68.7 Mm^3^. The depression storage of subbasin 127 is typified by sharp increases and gradual, rather than sharp (MS1), decreases during wet and dry periods (Figure 4b), respectively. Figure 4 demonstrates the impacts of considering/neglecting depressions in depression-dominated regions. The comparison between MS1 and MS2 shows that although ignoring depressions in dendritic basins might not considerably affect the amount of generated surface runoff (Figure 4a), the amount of water stored in depressions in depression-dominated basins cannot be neglected (Figure 4b). Particularly, incorporating depressions into a hydrologic model improves the physically-based simulations of hydrologic processes such as surface runoff and depression storage variations. For the depression-dominated RRB, MS2 provided more realistic results and hence it was selected to evaluate the effects of different calibration schemes (CS1 and CS2) on the SWAT modeling.

### 3.3. How Does the Separation of Wet and Dry Years Improve Water Quantity Modeling?

**Figure 5:**
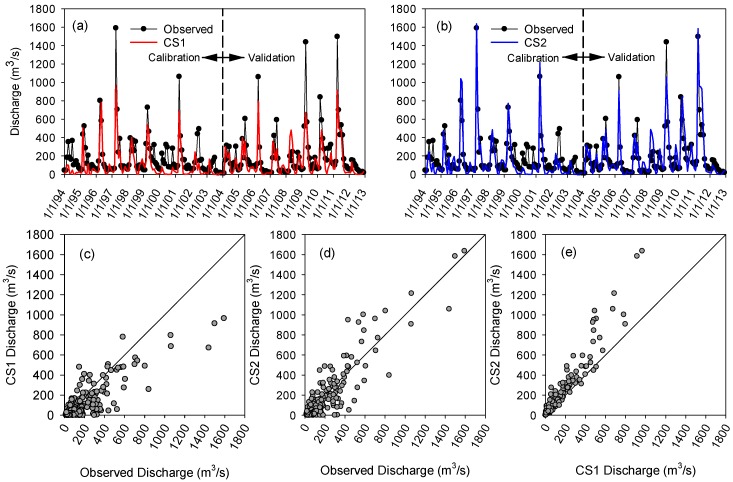
Observed versus simulated monthly discharges at the Grand Forks gauging station for (**a**) calibration scheme CS1 (traditional calibration scheme); (**b**) calibration scheme CS2 (wet-dry calibration scheme); (**c**–**e**) graphical comparisons of the observed and simulated discharges for CS1 and CS2.

To evaluate the performance of the two calibration schemes (CS1 and CS2), their modeling results were compared by using graphical and statistical measures. The traditional calibration scheme (CS1) did not account for the intrinsic differences of wet and dry years, while the wet-dry calibration scheme (CS2) used the relative deviation percentage from the mean streamflow to separate the entire simulation period into wet and dry years. That is, each wet/dry year was calibrated and validated with a group of other wet/dry years. Figure 5 shows a sample set of graphical comparisons between the observed and simulated monthly discharges at the Grand Forks gauging station for CS1 and CS2. Figure 5a indicates that CS1 did not provide accurate estimates of peak flows for both calibration and validation periods. Other studies have also highlighted that the traditional calibration of the SWAT model may not be able to provide satisfactory simulations of peak flows in the RRB [48]. In contrast, if the wet and dry years were separated for model calibration (i.e., CS2), a significant improvement was achieved. A visual comparison between Figure 5a,b reveals that the peak flows simulated in CS2 were very close to the observed ones. This improvement is particularly notable for the historical flood years, such as 1997 and 2011. The observed monthly discharge in April 1997 was 1591.7 m^3^/s and the simulated discharges for CS1 and CS2 were 965 m^3^/s and 1637 m^3^/s, respectively (Figure 5a,b). In addition to the graphical comparison, statistical indices showed that CS2 outperformed CS1 in both wet and dry years. For example, the NSE coefficient for the discharge at the Grand Forks station in the wet years increased from 0.69 of CS1 to 0.93 of CS2. Similarly, the NSE coefficient for the dry years also showed a sharp increase from 0.37 to 0.61 for CS1 and CS2. These results pinpoint the importance of separating wet and dry years in the calibration process.

Figure 5c–e show the comparisons of the calibration results for CS1 and CS2. Figure 5c indicates that the simulated discharges in CS1 are consistently lower than the observed ones, especially for the high peak flows. However, this underestimation was significantly improved by separating the wet and dry years in CS2 (Figure 5d). Figure 5d indicates that separating the simulation period into wet and dry years created a well-balanced distribution of the simulated vs. observed discharges. Figure 5e highlights the difference in discharges between CS1 and CS2. From Figure 5e, it can be observed that the majority of the simulated discharges in CS1 are lower than those in CS2. This improvement in CS2 can be attributed to the implementation of the separate calibration processes for the wet and dry hydroclimatic conditions in CS2. That is, when the wet and dry years are not separated, the calibration process aims to achieve the middle-ground values of the calibrated parameters that satisfy a wide variety of flow conditions (i.e., both wet and dry) over the entire simulation period. However, when wet and dry years are separated, the calibration process results in two sets of calibration parameters: one for wet years and one for dry years. Reasonably, the CS2 calibration results were closer to the actual streamflow conditions.

To compare the efficiencies of the two calibration schemes for the four gauging stations in the RRB, the simulated discharge series in CS2 for the wet and dry years were combined into a single time series for each station and then compared with those in CS1 for both calibration and validation periods. Table 2 summarizes the calculated statistics (NSE and PBIAS) for the two calibration schemes for the four gauging stations. The results in Table 2 show that the NSE and PBIAS values for all stations were improved from CS1 to CS2. In particular, the average NSE for the calibration period increased by 30.4% from CS1 to CS2. Similarly, the average NSE for the validation period increased by 19.6%. The Fargo station experienced the highest improvement in its validation results: the NSE increased by 51.2% from 0.41 to 0.62 in CS1 and CS2, respectively. All other stations also experienced notable improvements. The majority of NSE and PBIAS values for CS2 fell into the suggested acceptable ranges [50,51], except for the PBIAS values for the Doran station and its NSE for the validation period. These statistics for the two calibration schemes confirm the graphical comparisons and suggest that separating the wet and dry years significantly improves the hydrologic modeling, especially for basins under the influence of extreme hydroclimatic conditions (wet and dry). Therefore, CS2 was selected to perform the calibration and validation of the nutrient (NO_3_-N) modeling.

### 3.4. Water Quality Modeling

**Figure 6:**
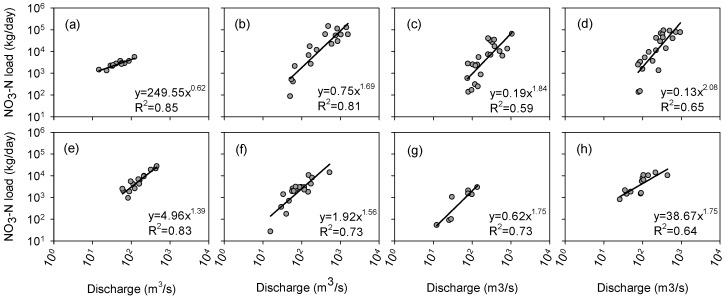
Relationships between the observed discharge and NO3-N load at the Grand Forks gauging station for different months from 2003 to 2017: (**a**) November, December, January, February, and March; (**b**) April; (**c**) May; (**d**) June; (**e**) July; (**f**) August; (**g**) September; and (**h**) October (R^2^ denotes the Pearson product-moment correlation coefficient).

Continuous daily time series of the observed water quality data were not available for the Red River and only some grab samples of NO_3_-N at the Grand Forks station were obtainable for a number of selected days over the course of 15 years from 2003 to 2017. Hence, a regression analysis was performed to establish the relationships between the river discharge and the NO_3_-N load for different months (Figure 6).

The variations of the NO_3_-N loads during the cold months in the RRB (i.e., November, December, January, February, and March) were not significant and fewer samples were available for these months. Therefore, one power relationship was considered for these months (Figure 6a). At the beginning of the growing season (April–June), however, the variation and magnitude of NO_3_-N loads were much higher, which can be directly attributed to the applications of fertilizers in the agricultural fields in the RRB (Figure 6b–d). After the initial months of the growing season, the NO_3_-N loads started becoming smaller in both range and magnitude (Figure 6e–h). The calculated Pearson product-moment correlation coefficient values for different months indicated that even for the months with smaller sample sizes, the fitted lines for discharge and NO_3_-N load were acceptable (R^2^ > 0.5 in Figure 6a–h). Eventually, these fitted power equations for different months were utilized to generate daily and monthly time series of NO_3_-N loads for the 2003–2012 period based on the corresponding discharge data.

**Figure 7:**
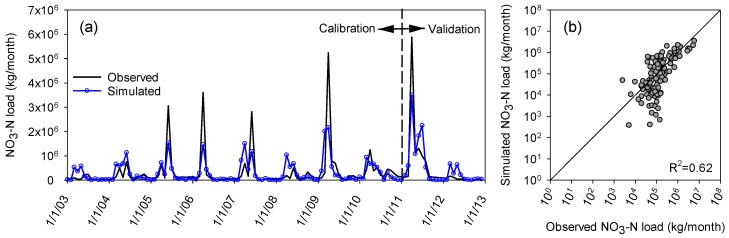
(**a**) Comparison of the simulated and observed chemographs and (**b**) graphical comparison of the observed and simulated NO_3_-N loads for CS2 at the Grand Forks gauging station.

The calibration and validation of water quality modeling were based on the results from CS2 and the observed NO_3_-N loading data at the Grand Forks gauging station. Because the NO_3_-N loading data were not available for 1994–2003, the periods of 2003–2010 and 2011–2012 were respectively selected for calibration and validation of the water quality modeling. Figure 7 shows the comparison of the observed and simulated NO_3_-N loads for CS2. Figure 7a suggests that except for the loading peaks associated with the flood peaks (note that water quality data during the floods were not available), the water quality simulations over the 10-year period are acceptable. Figure 7b depicts a graphical comparison between the observed and simulated NO_3_-N loads. The results also indicate that CS2 provided better estimations for the wet years than the dry years. The NSE values for the wet and dry years during the entire simulation period were 0.61 and 0.52, respectively, both of which are within the suggested acceptable range [50,51]. In addition to NSE, the PBIAS results for the wet and dry years (16.2% and −5.9%, respectively) were also within the acceptable range. The positive PBIAS for the wet years indicated that the model underestimated the NO_3_-N loads for the wet years, while the negative PBIAS for the dry years was indicative of a slight overestimation. The slight overestimation in the dry years and the underestimation in the wet years can be attributed to the use of the regression method as a substitute of real daily NO_3_-N loads due to the lack of the observed water quality data. The overall NSE and PBIAS values for the calibration and validation periods were 0.50 and 9.17; and 0.74 and −2.12, respectively. These results highlight the improvement from CS2 in simulating the NO_3_-N loads for both wet and dry years.

## 4. Discussion

Similar to other SWAT studies for depression-dominated areas, 25 SWAT parameters were selected for sensitivity analysis [17,28,48]. The most sensitive water quantity and quality parameters were selected for evaluating the impacts of different calibration schemes. These parameters are related to different processes considered in SWAT and are listed in Table 3. The detailed analysis of these parameters and their influences on CS1 and CS2 can shed light on the model performance in different schemes.

In SWAT, CN2 denotes the initial curve number for the antecedent moisture condition II. CN2 has a direct impact on the simulation of surface runoff and is a function of soil type, land use and land cover, soil moisture, and other conditions. The CN2 calibration results for the two schemes highlight the importance of the CN2 in wet and dry years. The results suggest that when the CN2-change increased between 0.31% and 8.37%, the simulated discharges for the wet years were close to the observed ones. This increase was required to be able to capture the peaks during the wet years. However, the calibrated range of CN2 for the dry years indicated an opposite trend. The best model performance occurred when the CN2-change decreased up to 6.45% or slightly increased by 2.28%. In contrast to the CS2 results, the CS1 resulted in a much wider range of CN2 variations (i.e., from −16.59% to 12.59%), suggesting that in order to better simulate discharge, CN2 changes need to be increased or decreased up to 12.59% or 16.59%, respectively. Reasonably, dividing the simulation time span into the wet and dry years provided more specific ranges that were in accordance with the actual hydroclimatic conditions of the wet and dry years. As discussed before, incorporating depressions in the SWAT model significantly improved the simulation of hydrologic processes. It was found that the hydraulic conductivity of the incorporated depressions (WET_K) was one of the more sensitive parameters. The CS2 results indicated that WET_K for the wet years was greater than that of dry years. Particularly, when there was more water in depressions in the wet years, the hydraulic conductivity of the depressions was higher. In CS1, however, the calibration range was a combination of both low and high WET_K values, which was one of the factors that affected the model performance in CS1.

In addition to CN2 and WET_K, three parameters related to the snow processes were found to be sensitive. This is not surprising, since the RRB is a typical cold-climate basin. Table 3 shows that the snowmelt temperature varies significantly between the wet and dry years. The CS2 results indicate that the calibrated ranges of the snow melt temperature (SMTMP) are from 2.74 to 4.87 °C and from −0.2 to 1.34 °C in the wet and dry years, respectively. The higher SMTMP in the wet years can be attributed to the hydrologic and meteorological characteristics of the RRB. In the wet years, early spring rainfall events coincide with the snowmelt process and the frozen soil condition, causing extreme streamflow conditions. The synchronization of these hydroclimatic processes has been found to be one of the major causes of major historical floods in the RRB [37,54]. Therefore, to simulate the snowmelt process in early springs, CS2 accounted for higher SMTMP in the wet years. The results also indicated that the SMTMP range for CS1 was closer to that of CS2 for the wet years than the dry years, implying that CS1 could not provide accurate estimates of snowmelt for the dry years. In addition, the maximum and minimum snowmelt factors (SMFMX and SMFMN) were subject to the changes from wet to dry hydroclimatic condition. Both SMFMX and SMFMN had greater values in the wet years due to the greater snowmelt and the resulting peak flows in early springs.

Soil parameters were also important in different calibration schemes. ESCO is a coefficient in SWAT that denotes the depth of water evaporated from the soil. When ESCO decreases, the model takes more water from the lower soil layers [40]. The ranges of the calibrated ESCO for the wet and dry years are in agreement with what is expected. The ESCO values for the dry and wet years varied from 0.05 to 0.15 and from 0.42 to 0.64, respectively, indicating that more water was extracted from the deep soil layers to satisfy the evaporative demand in the dry years, whereas the wet years accounted for a smaller amount of water evaporated from the deep soil layers. The plant available water in SWAT was represented by SOL_AWC. Table 3 suggests that the simulations for the wet years in CS2 were closer to the observations when SOL_AWC increased in a range from 21.97% to 31.15%. That is, in the wet years more water was available for plants. For the dry years, however, the acceptable model performance was achieved if SOL_AWC changed within the range from −12.71% to 8.25%. Comparing these two SOL_AWC ranges for the wet and dry years reveals the tendency of CS2 to have less water available for plants in the dry years and more water available for plants in the wet years. Similar to the CN2 variations in CS1, the calibrated range of SOL_AWC for CS1 is comprised of a wider range to cover the mixed characteristics of both wet and dry years.

ALPHA_BF and GW_REVAP are two sensitive parameters associated with the groundwater processes in SWAT. ALPHA_BF is a baseflow recession constant that controls the response of the groundwater system to changes in recharge and GW_REVAP handles the movement of water from the shallow aquifer to the unsaturated zone through the capillary fringe [40]. It has been suggested that ALPHA_BF values between 0.1 and 0.3 correspond to the regions with a slow groundwater response whereas ALPHA_BF values between 0.9 and 1 are associated with the regions with quick groundwater response [40]. The results from CS2 show that the RRB tends to have a medium to fast groundwater response in the wet years, while the groundwater response in the dry years was slow. Specifically, the calibrated ALPHA_BF values ranged from 0.79 to 0.91 and from 0.06 to 0.38 for the wet and dry years, respectively. These results are in accordance with Lin et al. [28], in which they suggested the ALPHA_BF range of 0.01 to 0.95 for the RRB. For GW_REVAP, when the unsaturated zone is dry, more water leaves the water table aquifer through the capillary fringe (i.e., higher GW_REVAP). This process was captured in CS2, where the dry years had greater values of GW_REVAP and the wet years had smaller values of GW_REVAP in their ranges (Table 3).

In addition to water quantity parameters, four water quality parameters were found to be important in the calibration of modeling the NO_3_-N loads. Parameters BC1 and BC2 directly affect the NO_3_-N concentrations and BC3 and RS3 have indirect impacts. The conversion of ammonia to NO_3_-N is an important process that is simulated in SWAT via a rate constant for biological oxidation of ammonia (i.e., BC1). The concentration of NO_3_-N in streams also increases due to the oxidation of nitrite and decreases by algae uptake. The variations of NO_3_-N in streams can also be controlled by the rate constant for biological oxidation of nitrite to nitrate (BC2), which is a function of the in-stream oxygen concentration. However, the local rate constant for the hydrolysis of organic nitrogen to ammonium (BC3) and benthos source rate for ammonium nitrogen (RS3) influence the concentration of ammonium, and hence indirectly affect NO_3_-N. Table 3 indicates that the calibrated values of these water quality parameters for the wet years are slightly higher than those for the dry years. The most significant difference between the wet and dry years can be observed for BC1. The recommended ranges of BC1 for the wet and dry years are 0.50–0.60 and 0.10–0.11, respectively. Since the wet years in the RRB are associated with heavy rainfalls during the growing season, more nutrients are washed out of the agricultural fields through surface runoff and soil erosion, which results in a higher load of nutrients in the stream. These processes were reflected in the modeling results from CS2 for the wet and dry years.

## 5. Conclusions

This study was aimed to improve the SWAT water quantity and quality modeling by incorporating the impacts of surface depression and employing a wet-dry calibration scheme. In the application within the RRB, surface depressions were delineated by using a DEM-based delineation method. The identified depressions were incorporated into subbasins via the wetland feature of SWAT. A simulation period from 1989 to 2012 was selected for the SWAT modeling and the required input data were obtained from various sources. The impacts of depressions on the modeling of hydrologic processes were assessed by two modeling scenarios (MS1 and MS2). In addition, to highlight the influence of varying hydroclimatic conditions (e.g., extreme wet and dry conditions), two calibration schemes (CS1 and CS2) were implemented. In CS2, calibration and validation were conducted separately for the wet and dry years that were determined for four USGS gauging stations based on their relative deviation percentage from the mean discharge. Finally, the simulation results were evaluated by using the graphical method and two statistics: NSE and PBIAS.

The results highlighted that neglecting the role of surface depressions in the modeling of depression-dominated basins may result in unrealistic surface runoff simulations. The calibration results indicated that the wet-dry calibration scheme CS2 significantly improved the simulations of discharges at all four stations by considering the distinct hydroclimatic conditions in the wet and dry years. Specifically, the average NSE in CS2 was improved by 30.4% and 19.6% for the calibration and validation periods, respectively. Both NSE and PBIAS fell into the suggested acceptable ranges. With the NSE values of 0.50 and 0.74 for the calibration and validation, respectively, the simulations of NO_3_-N loads also showed good agreement with the observed data, although the model slightly underestimated the NO_3_-N loads for the wet years and overestimated the NO_3_-N loads for the dry years. This study highlights the importance of accounting for the impacts of topographic depressions in hydrologic and water quality modeling, especially under varying hydroclimatic conditions. The proposed methodology for separate wet-dry calibrations can be applied to other basins with a similar hydrologic regime.

## Figures and Tables

**Table 1:** Wet and dry years for four gauging stations along the Red River.

Station	Wet Years	Dry Years
**Drayton**	1996, 1997, 1999, 2001, 2005, 2009, 2010, 2011	1994, 1995, 1998, 2000, 2002, 2003, 2004, 2006, 2007, 2008, 2012
**Grand Forks**	1997, 1999, 2001, 2009, 2010, 2011	1994, 1995, 1996, 1998, 2000, 2002, 2003, 2004, 2005, 2006, 2007, 2008, 2012
**Fargo**	1997, 1998, 2001, 2005, 2007, 2009, 2010, 2011	1994, 1995, 1996, 1999, 2000, 2002, 2003, 2004, 2006, 2008, 2012
**Doran**	1995, 1997, 2001, 2005, 2006, 2007, 2009, 2010, 2011	1994, 1996, 1998, 1999, 2000, 2002, 2003, 2004, 2008, 2012

**Table 2:** Model performance statistics (Nash–Sutcliffe efficiency (NSE) and percent bias (PBIAS)) for the two calibration schemes (CS1: traditional calibration scheme and CS2: wet-dry calibration scheme).

Station		CS1		CS2	
		NSE	PBIAS (%)	NSE	PBIAS (%)
**Drayton**	Calibration	0.55	24.86	0.62	11.95
	Validation	0.65	15.01	0.73	−4.33
**Grand Forks**	Calibration	0.55	24	0.71	11.81
	Validation	0.67	14.94	0.77	−0.59
**Fargo**	Calibration	0.51	21.8	0.70	7.25
	Validation	0.41	26.27	0.62	12.97
**Doran**	Calibration	0.40	28.53	0.57	56.28
	Validation	−0.04	44.83	−0.04	34.17

**Table 3:** Water quantity and quality parameters for different calibration schemes (CS1: traditional calibration scheme; CS2: wet-dry calibration scheme).

Parameter *	Process	Unit	Initial Range	CS1	CS2	
					Wet	Dry
CN2	Surface runoff	% change	[−20, 20]	[−16.59, 12.59]	[0.31, 8.37]	[−6.45, 2.28]
ALPHA_BF	Groundwater	1/day	[0, 1]	[0.01, 0.68]	[0.79, 0.91]	[0.06, 0.38]
SOL_AWC	Soil water	% change	[−40, 40]	[−18.53, 24.54]	[21.97, 37.15]	[−12.71, 8.25]
GW_REVAP	Groundwater	-	[0.02, 0.2]	[0.18, 0.20]	[0.04, 0.11]	[0.07, 0.16]
SMTMP	Snow	°C	[−5, 5]	[1.72, 4.92]	[2.74, 4.87]	[−0.20, 1.34]
SMFMX	Snow	mm/day-°C	[0, 10]	[2.41, 6.41]	[3.71, 6.64]	[3.10, 6.04]
SMFMN	Snow	mm/day-°C	[0, 10]	[−1.23, 5.01]	[1.78, 4.52]	[0.01, 1.54]
ESCO	Soil evaporation	-	[0.01, 1]	[0.11, 0.37]	[0.42, 0.64]	[0.05, 0.15]
WET_K	Wetlands	mm/h	[0, 1]	[0.29, 0.92]	[0.79, 0.94]	[0.20, 0.67]
RS3	Water quality	mg/(m²day)	[0, 1]	-	[0.05, 0.15]	[0.05, 0.10]
BC1	Water quality	1/day	[0.1, 1]	-	[0.50, 0.60]	[0.10, 0.11]
BC2	Water quality	1/day	[0.2, 2]	-	[0.20, 0.30]	[0.20, 0.22]
BC3	Water quality	1/day	[0.2, 0.4]	-	[0.20, 0.22]	[0.20, 0.22]

* CN2 = curve number, ALPHA_BF = baseflow recession constant, SOL_AWC = available water capacity, GW_REVAP = revap coefficient, SMTMP = threshold temperature for snowmelt, SMFMX = maximum melt factor, SMFMN = minimum melt factor, ESCO = soil evaporation compensation coefficient, WET_K = effective saturated hydraulic conductivity of the wetlands, RS3 = sediment source rate for ammonium nitrogen, BC1 = rate constant for oxidation of ammonium nitrogen, BC2 = rate constant for biological oxidation of nitrate to nitrate, and BC3 = local rate constant for hydrolysis of organic nitrogen.
